# Maternal Group B Streptococcal Rectovaginal Colonization after Intrapartum Antibiotic Prophylaxis

**DOI:** 10.3390/children9121848

**Published:** 2022-11-28

**Authors:** Ping Liu, Qiaoli Feng, Yiheng Liang, Xinxin Wang, Zhansong Xiao, Liting Huang, Yun Li, Yuqing Deng, Lin Yu, Yang Xin, Shangrong Fan

**Affiliations:** 1 Department of Obstetrics and Gynecology, Peking University Shenzhen Hospital, Shenzhen 518036, China; 2 Shenzhen Key Laboratory on Technology for Early Diagnosis of Major Gynecological Diseases, Shenzhen 518036, China

**Keywords:** colonization, group b streptococcus, intrapartum antibiotic prophylaxis

## Abstract

Maternal rectovaginal colonization with Group B Streptococcus (GBS) during labor is a prerequisite for neonatal early-onset GBS disease. Intrapartum antibiotic prophylaxis (IAP) has been proven to prevent GBS perinatal infection, while there are few studies on the evaluation of the effectiveness of different antibiotic prophylaxis regimens. This study aimed to assess the maternal rectovaginal GBS colonization status after IAP, antimicrobial susceptibility and maternal and neonatal outcomes among women administered different antibiotic prophylaxis regimens. A prospective study was conducted between June 2018 and June 2022. GBS carriers identified at 35–37 weeks of gestation were provided IAP (penicillin, cefazolin or clindamycin) at delivery based on the local protocol for GBS prevention. Rectovaginal samples were obtained from participants again after delivery. Antimicrobial susceptibility testing in GBS isolates was performed using the broth microdilution method. A total of 295 cases were included in this study. In the postpartum re-examination for GBS, the overall negative rectovaginal culture rate was 90.8% (268/295). Women who received cefazolin prophylaxis had the highest negative culture rate (95.2%, 197/207), which was followed by those who received penicillin (80.7%, 67/83) and clindamycin (80.0%, 4/5) (*p* = 0.001). All GBS isolates achieved sensitivity to penicillin and cefazolin, whereas resistance to clindamycin was shown in 21.4% of the strains. There were no significant differences in maternal and neonatal outcomes among the IAP groups. The use of IAP is highly effective in reducing the maternal rectovaginal GBS colonization. Cefazolin may offer equivalent efficacy and safety compared to standard penicillin prophylaxis.

## 1. Introduction

Group B Streptococcus (GBS), or Streptococcus agalactiae, is a Gram-positive bacterium that colonizes 11–35% of pregnant women at vaginal/rectal sites [1]. Maternal GBS colonization is an important risk factor for perinatal infection in neonates [2,3]. Invasive GBS disease (0.41 per 1000 live births) is associated with a high mortality rate of almost 50% in newborn infants [2,3]. Infant survivors of invasive GBS sepsis had 3.5-fold greater odds of neurological impairment by 1 year of age [4]. GBS may also cause maternal urinary tract infection, intraamniotic infection or endometritis and is associated with preterm labor and stillbirth [3,5,6,7,8].

Universal screening for GBS in late pregnancy and providing intrapartum antibiotic prophylaxis (IAP) for GBS carriers to prevent neonatal GBS infection are recommended by international societies [2,3]. The first-line antimicrobial prophylaxis against GBS infection is penicillin or amoxicillin, which have been deemed equally effective [2]. For women who have penicillin allergies and a low risk of anaphylaxis, intravenous cefazolin is recommended [2,5,9,10,11]. For women allergic to penicillin with a history of severe reactions, the use of clindamycin or vancomycin is preferable [5]. However, recent studies have reported increasing antimicrobial resistance during GBS prevention [12,13,14,15]. Hayes et al. reviewed antibiotic resistance in GBS prevention and reported that GBS is universally susceptible to beta-lactam antibiotics [14], whereas there have been reports of reduced susceptibility to beta-lactams, including penicillin, in some countries [16,17]. Resistance to second-line antibiotics, such as erythromycin and clindamycin, remains high among GBS, with several countries noting increased resistance rates in recent years [14].

Along with the appearance of nonsusceptible strains for erythromycin and clindamycin, IAP failure is becoming an important issue for managing women with GBS colonization. In addition, IAP policies vary in different countries and regions, and standard penicillin may be unavailable in some regions or cannot be provided in a timely manner in emergency situations due to the request for skin tests. Therefore, the evaluation of the effectiveness and safety of different antibiotic prophylaxis regimens is needed for IAP implementation. The present study aimed to assess the maternal rectovaginal GBS colonization status after IAP as well as maternal and neonatal outcomes among women administered different antibiotics.

## 2. Materials and Methods

### 2.1. Study Design and Participants

This was a prospective study of women with singleton pregnancies with GBS colonization diagnosed by rectovaginal culture at 35–37 weeks of gestation and admitted to Peking University Shenzhen Hospital for delivery between June 2018 and June 2022. We excluded women with planned caesarean section or deliveries before 37 weeks of gestation to eliminate the bias of adverse outcomes due to early gestational age. Approval for this study was obtained from the Ethics Committee of the Peking University Shenzhen Hospital (Ref. No. 201806).

### 2.2. GBS Culture

Universal GBS screening at 35–37 weeks of gestation and re-examination after IAP were performed in all participants by rectovaginal culture to detect GBS status. Separate swabs were collected from the lower vagina without the use of a speculum and then the rectum. The specimens were transferred into a selective enrichment broth and incubated for 24–48 h at 37 °C in 5% CO_2_ and then transferred to a blood agar plate for subculture. After an overnight incubation period, the plates were analyzed for GBS colonies [3].

### 2.3. Antimicrobial Susceptibility Testing

Forty-five GBS isolates from randomly selected participants were tested for antimicrobial susceptibility to ampicillin, penicillin, clindamycin, erythromycin, vancomycin, levofloxacin, quinupristin-dalfopristin, tetracycline and linezolid using the broth microdilution method recommended by The Clinical and Laboratory Standards Institute (CLSI) [18]. The minimum inhibitory concentration (MIC) breakpoints for categories including “sensitivity” (S), “intermediate sensitivity” (I), and “resistant” (R) referred to the CLSI criteria [16], while breakpoints for cefazolin, cefepime, ceftibuten and ceftizoxime are not provided by CLSI, as penicillin is regarded as a surrogate for the antimicrobials listed above.

### 2.4. Intrapartum Antibiotic Prophylaxis

Penicillin G (penicillin group) is the first choice for patients with a negative penicillin skin test. The initial dose of 4.8 million units is intravenously infused, and then, 2.4 million units are intravenously infused every 4 h until delivery. Women with a low risk of penicillin allergy (no history of allergic reactions, angioedema, respiratory distress or urticaria after penicillin or cephalosporin use) were administered cefazolin (cefazolin group), with an initial dose of 2 g given intravenously, and then, 1 g was given intravenously every 8 h until delivery. Women with a high risk for anaphylaxis (a history of allergic reactions, angioedema, respiratory distress or urticaria after penicillin or cephalosporin use) were administered clindamycin (clindamycin group), with 0.9 g given intravenously every 8 h until delivery. For women who had a cervical ripening balloon placed (Cook Cervical Ripening Balloon, Cook Medical Europe, Ireland) due to unfavorable cervix, antibiotic prophylaxis was administered 1–2 h before the cervical balloon was placed, with another single dose intravenously infused after placement. Prophylactic antibiotics in subsequent dosage were administered from the time of labor onset or premature rupture of the membranes until delivery.

### 2.5. Data Collection

In this study, data on maternal history (including age, parity, method of conception, gestational age at the time of GBS screening, gestational diabetes mellitus and history of hypertension), information on IAP (type and doses) and pregnancy outcomes (gestational age at delivery, mode of delivery, maternal and neonatal complications such as prelabor rupture of membranes, intrauterine infection, chorioamnionitis, low birth weight, neonatal intensive care unit (NICU) admission, low Apgar score and neonatal early-onset sepsis) were recorded in a secure electronic database.

### 2.6. Statistical Analysis

Descriptive data are presented as the mean and standard deviation (SD) for continuous variables and as counts and percentages for categorical variables. Comparisons between groups were performed by the χ^2^ test or Fisher’s exact test for categorical variables and one-way ANOVA for continuous variables, with post hoc Bonferroni correction. A two-tailed *p* value < 0.05 was considered statistically significant for comparisons among the three groups, and a *p* value < 0.017 was considered statistically significant for post hoc Bonferroni correction. Data analyses were performed using the statistical software package SPSS 23.0 (SPSS Inc., Chicago, IL, USA).

## 3. Results

### 3.1. Sociodemographic and Clinical Characteristics

**Figure 1:**
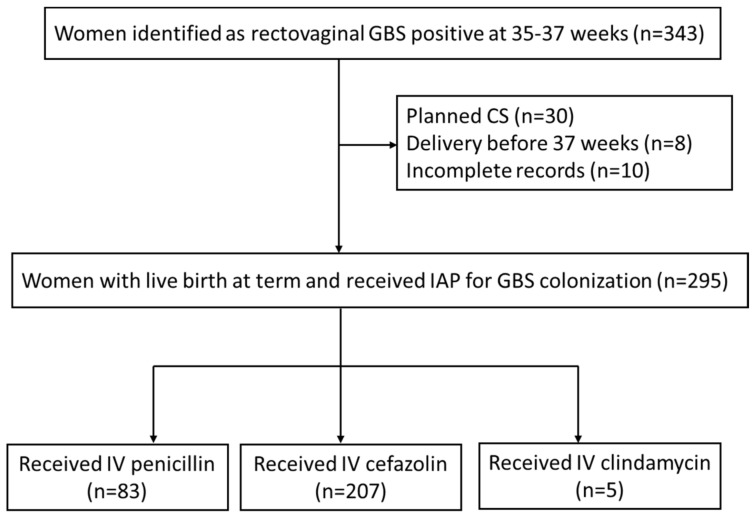
Flow chart of study population selection.

A total of 343 pregnant women who had positive rectovaginal GBS cultures at 35–37 weeks of gestation and underwent re-examination after IAP were enrolled in this study. Forty-eight (14.0%) patients were excluded due to planned caesarean section (n = 30), delivery before 37 weeks of gestation (n = 8) and incomplete records (n = 10). The remaining 295 patients were included in the analysis, consisting of 83 patients (28.1%) receiving penicillin, 207 patients (70.2%) receiving cefazolin and 5 patients (1.7%) receiving clindamycin during delivery (Figure 1). There were 25 patients (8.5%) who had a cervical ripening balloon placed for labor induction.

The maternal demographic characteristics of the whole study population and each IAP group are presented in Table 1. There were no significant differences in parity, the type of conception, gestational age at screening and delivery, or the prevalence of hypertension or gestational diabetes mellitus among women receiving penicillin, cefazolin or clindamycin for IAP, except that women receiving clindamycin were older than those receiving cefazolin (*p* = 0.015).

### 3.2. Maternal GBS Colonization after IAP

In the postpartum re-examination for GBS, the overall negative rectovaginal culture rate was 90.8% (268/295). Women who received cefazolin prophylaxis had the highest negative culture rate (95.2%, 197/207), which was followed by those who received penicillin (80.7%, 67/83) and clindamycin (80.0%, 4/5) (*p* = 0.001) (Table 2). In the subgroup analysis, the cefazolin group even achieved a better negative culture rate than the penicillin group (*p* < 0.017). As the duration of the labor process was different between cases, the negative culture rates of postpartum GBS re-examination based on dosage in different types of IAP are presented and compared in Table 2. There were no significant differences in the negative rates of postpartum GBS cultures between women who received a single dose of IAP and those who received two doses or more, regardless of whether they were in the penicillin, cefazolin or clindamycin groups (all *p* > 0.05). For women who had a cervical ripening balloon placed for labor induction, maternal negative GBS colonization after IAP was comparable with those without mechanical cervical ripening (88.0% vs. 91.1%, *p* = 0.878). Similar findings were detected in the penicillin, cefazolin and clindamycin groups, with *p* values of 0.441, 0.365 and 0.600, respectively.

### 3.3. Antimicrobial Susceptibility of GBS from Pregnant Women

All GBS isolates were sensitive to penicillin and ampicillin, with an MIC range below 0.25 µg/mL, which achieved an inhibitory concentration of 90% of the strains. According to the recommendation of the CLSI, penicillin-susceptible GBS are also susceptible to cefazolin [18]. Ten percent of the isolates demonstrated intermediate sensitivity to erythromycin (MIC50 ≤ 0.25 µg/mL), and 40% were resistant. A total of 21.4% of the isolates showed resistance to clindamycin (Table 3).

### 3.4. Maternal and Neonatal Outcomes among the IAP Groups

The maternal and neonatal outcomes of our study population are shown in Table 4. The majority of women (279/295, 94.6%) had vaginal delivery, whereas 16 women (5.4%) had emergency caesarean sections due to fetal distress or no progress in labor. The prevalence of prelabor rupture of membranes, intrauterine infection and chorioamnionitis was 24.1%, 0.7% and 3.1%, respectively. Most neonates were born in good condition, except that 4 (1.4%) had low birth weight, 16 (5.4%) were admitted to the neonatal intensive care unit due to severe complications, one (0.3%) had a low 5 min Apgar score and one (0.3%) had neonatal early-onset sepsis. There were no significant differences in maternal and neonatal outcomes among the IAP groups. There were no severe side effects of any IAP administration during the study period.

### 3.5. Neonatal GBS Infections during the Study Period

One infant presented with GBS culture-proven sepsis during our study period. The infant’s mother was diagnosed with gestational diabetes through an oral glucose tolerance test (5.02–9.58–9.42 mmol/L) and screened positive by rectovaginal culture for GBS at 35 weeks of gestation. The male infant weighed 3100 g and was born via spontaneous vaginal delivery at 38 weeks of gestation but did not receive IAP in a timely manner due to precipitate labor. The infant’s Apgar scores were 9 and 10 at 1 and 5 min, respectively.

**Figure 2:**
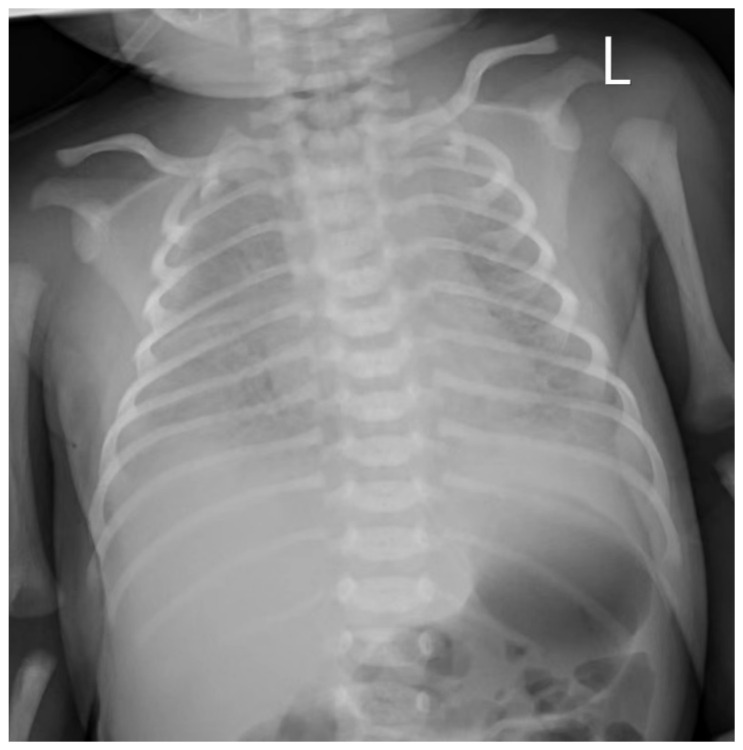
Chest radiography of neonates with GBS sepsis: significantly decreased bilateral transparency and extensive granular high-density shadows, with blurred bilateral diaphragmatic surfaces and costodiaphragmatic angles. L, left side of the neonate.

The newborn infant developed dyspnea and groan after two hours of life, with a blood pressure that dropped to 59/37 mmHg, and was transferred to the neonatal intensive care unit. The infant required continuous positive airway pressure (CPAP)-assisted ventilation and was given penicillin combined with meropenem after a blood culture was obtained. Chest radiography demonstrated significantly decreased bilateral transparency and extensive granular high-density shadows, with blurred bilateral diaphragmatic surfaces and costodiaphragmatic angles (Figure 2). Coffee-like substances were found in the gastric tube, and bloody sputum was detected in the tracheal tube. The transcutaneous oxygen saturation was maintained at approximately 88–90% under oxygen inhalation. The blood culture was reported to be positive for GBS, which was susceptible to penicillin, ampicillin, levofloxacin, moxifloxacin, linezolid, vancomycin, teicoplanin, tegacyclin and compound sulfamethoxazole. Intravenous meropenem (0.12 g every 12 h for 7 days and then 0.06 g every 8 h) and vancomycin (0.04 g every 12 h) were given to treat infection, fluconazole (18 mg once) was administered for fungal prevention, and continuous dopamine (45 mg) combined with dobutamine (45 mg) was given as a vasopressor for shock therapy. A repeat blood culture obtained 72 h after delivery was negative. Cerebrospinal fluid obtained 72 h after delivery showed no pleocytosis, and the culture was negative. Intravenous meropenem and vancomycin were continued for a total of 14 days. The infant was discharged home on Day 19.

## 4. Discussion

The significant effect of IAP for GBS-colonized women to prevent early-onset neonatal GBS infection has been proven by large cohort studies [19,20,21] and is currently a standard of care in many guidelines [2,19,22,23,24,25,26]. Ampicillin IAP decreases maternal vaginal colonization and prevents neonatal surface colonization in 97% of cases if IAP is administered at least 2 h before delivery [27]. In China, IAP administration in women with GBS colonization reduces GBS neonatal sepsis by 72–75% [28]. However, there is currently a lack of comparative studies evaluating the clinical efficacy of IAP according to the different types of intrapartum antibiotic regimens for the prevention of neonatal GBS disease. This study reviewed the efficacy of IAP in pregnant women with GBS colonization who presented to Peking University Shenzhen Hospital and demonstrated that intrapartum antibiotics significantly decrease maternal rectovaginal GBS colonization, especially cefazolin, which even achieves a higher negative GBS culture rate than penicillin and clindamycin. A single dose of antimicrobial prophylaxis shows a comparable effect with multiple doses in reducing GBS colonization. Mechanical cervical ripening for the induction of labor does not affect the efficacy of IAP in women with GBS colonization. All GBS isolates achieved sensitivity to penicillin and cefazolin, whereas resistance to clindamycin was shown in 21.4% of the strains. There were no significant differences in maternal and neonatal outcomes among women who received penicillin, cefazolin and clindamycin as IAP regimens for GBS.

Intravenous penicillin remains the first-line agent for intrapartum prophylaxis due to its targeted spectrum of antimicrobial activity against Gram-positive bacteria and low likelihood of inducing resistance in other microorganisms [2]. Cefazolin and clindamycin are acceptable alternatives for GBS prophylaxis in women with a penicillin allergy. In our study, the overall negative rectovaginal culture rate was 90.8%, which is similar to that in Scasso’s study (88%) [29], supporting the significant effect of IAP in the prevention of GBS colonization. Interestingly, in the subgroup analysis, women who received cefazolin prophylaxis had a significantly higher negative GBS culture rate (95.2%) than those who received penicillin (80.7%) and clindamycin (80.0%) (*p* = 0.001). The performance of penicillin may be underestimated, as the dosage of penicillin used in our study was lower than the recommended dosage in international guidelines due to the penicillin specifications in our hospital (initial dose of 4.8 million units followed by 2.4 million units every 4 h versus an initial dose of 5 million units followed by 3 million units every 4 h) [2]. Nonetheless, our findings support the significant effect of cefazolin, which has a lower risk of anaphylaxis and is more convenient in clinical practice without the requirement of skin tests.

In previous studies, IAP administration for at least 4 h was considered an adequate IAP regimen for GBS prevention [3,9,10]. However, prophylaxis provided for a shorter duration of 30 min to 2 h has been proven to have a high serum antibiotic concentration that far exceeds the minimum inhibitory concentration against GBS [11]. In this study, we did not find a significant difference in the reduction in maternal colonization between single dose and multiple doses of IAP, regardless of the type of antibiotic regimen, which may be the result of the fact that the duration of IAP before delivery in all participants was at least 2 h. Such observations support that at least one IAP dose prior to delivery might still yield benefits in precipitous delivery. The rapid effect of IAP in reducing vaginal GBS colony counts has also been detected in another study involving 27 women with positive intrapartum vaginal cultures (200- and 10,000-fold decline within 2 h and 4 h prior to delivery, respectively) [30].

In this study, we also explored the influence of cervical ripening balloon placement, which might increase the risk of ascending infection, and we did not find a significant difference in the IAP effect between women with and without cervical ripening balloon placement for labor induction. However, the standard protocol of IAP in women induced by cervical ripening balloon still needs further investigation in large prospective cohorts.

Antibiotic resistance in GBS strains is a concern in the prevention of GBS invasive disease. The findings from recent studies support the increasing resistance to antibiotics used in GBS treatment [12,13,14,15], especially erythromycin and clindamycin, the second-line antibiotics recommended in cases of severe penicillin allergies, which have resistance rates of 21.3–74.1% in different regions [21,23,31,32,33,34,35,36,37]. The results of our study also demonstrated high resistance to erythromycin and clindamycin (40.0% and 21.4%, respectively). Owing to the high and increasing resistance, the Royal College of Obstetricians and Gynecologists (RCOG) no longer recommends the use of clindamycin in UK-based guidelines [23]. Resistance to penicillin is relatively rare [21,38], which has also been supported by evidence from our study, in which all GBS isolates were susceptible to penicillin. Although we did not assess the susceptibility of GBS to cefazolin in this study, penicillin-susceptible GBS are also considered susceptible to cefazolin according to the recommendations from the CLSI [18], as they are both beta-lactam antibiotics. Fiore Mitchell and colleagues have tried to explore the maternal and transplacental pharmacokinetics of cefazolin and found that cefazolin concentrations greater than or equal to the MIC90 for GBS were attained in all maternal, fetal, and amniotic fluid samples 30 min after the completion of cefazolin infusion [39].

For the maternal and neonatal outcomes analyzed, no statistically significant difference was found among women who received penicillin, cefazolin and clindamycin. The comparable findings between cefazolin and penicillin are consistent with the results reported by Antonello’s team [40], which indicates that cefazolin can be an optimal choice to offer safety in the use of this antibiotic in situations where penicillin is contraindicated or unavailable. During our study period, there was one GBS patient who screened positive, did not receive IAP in a timely manner and developed neonatal GBS sepsis and septic shock. This case further supports the importance of IAP in pregnant women with GBS colonization to prevent adverse pregnancy outcomes.

Recently, cefazolin has been suggested as a promising prophylactic antibiotic for preventing the transmission of GBS due to its rare resistance to GBS and low frequency of cross-allergenicity to penicillin (ranging from 2% to 7%) [41]. The findings of our study that cefazolin achieved a higher negative rate of maternal GBS colonization after IAP and had maternal and fetal outcomes comparable to penicillin, together with previous evidence of a high cefazolin concentration greater than the MIC90 in maternal and fetal compartments within 18–30 min after drug administration [30,39], support the advantage of cefazolin as a reasonable and safe antibiotic prophylaxis regimen in clinical practice, especially in imminent delivery, as it does not require a skin test. Further investigation in randomized controlled trials for the therapeutic effect between penicillin and cefazolin is still needed.

The advantage of this study lies in the prospective nature to compare the efficacy of three types of IAP for GBS, especially the relatively large sample size of women who received cefazolin, which provides certain evidence for the clinical application of intrapartum antibiotics to prevent GBS infection in the future. This is also the first study to evaluate the changes in maternal GBS colonization among various IAP regimens. However, there are several limitations that should be considered when interpreting the results. First, because the number of patients in the clindamycin group was very small, the observations for clindamycin remain uncertain and need further investigation in larger studies. Second, this study was conducted in a single maternity unit and followed the local IAP protocol for the prevention of GBS, which is slightly different from the current international guidelines regarding the administered dosage of penicillin, leading to a potential deviation in comparisons with other studies. Last, the diagnosis of GBS colonization was based on a positive culture test in late pregnancy, and there was no intrapartum GBS examination to confirm GBS colonization before IAP, which may overestimate the actual number of patients with GBS colonization in this study. However, colonization in late pregnancy has been proven to be highly correlated with intrapartum colonization [2,42].

## 5. Conclusions

In conclusion, the use of IAP is highly effective in reducing maternal rectovaginal GBS colonization. Cefazolin may offer equivalent efficacy and safety compared to standard penicillin prophylaxis.

## Figures and Tables

**Table 1:** Maternal characteristics of the study population in the IAP groups.

Characteristics	Total	Penicillin (n = 83)	Cefazolin (n = 207)	Clindamycin (n = 5)	*p*
Maternal age (years)	30.6 (3.7)	31.9 (3.3)	30.3 (3.7)	34.4 (4.6)	0.009 ^a^
Parity					
Nulliparous	187 (63.4%)	58 (69.9%)	127 (61.4%)	2 (40.0%)	-
Multiparous	108 (36.6%)	25 (30.1%)	80 (38.6%)	3 (60.0%)	0.207
Conception					
Spontaneous	281 (95.3%)	77 (92.8%)	199 (96.1%)	5 (100.0%)	-
In vitro fertilization	14 (4.7%)	7 (7.2%)	8 (3.9%)	0	0.401
Gestational age at screening (weeks)	36.2 (0.7)	36.3 (0.7)	36.2 (0.7)	36.0 (0.7)	0.476
Gestational age at delivery (weeks)	39.5 (0.9)	39.7 (0.8)	39.4 (1.0)	40.0 (0.5)	0.069
Hypertension	4 (1.4%)	0	4 (1.9%)	0	0.609
Gestational diabetes mellitus	38 (12.9%)	15 (18.1%)	21 (10.1%)	2 (40.0%)	0.037 ^b^

Descriptive data were presented in mean and SD for continuous variables and in counts and percentages for categorical variables. ^a^, Post hoc comparisons between groups: penicillin vs cefazolin (*p* = 0.048), penicillin vs. clindamycin (*p* = 0.043), cefazolin vs. clindamycin (*p* = 0.015); ^b^, Post hoc comparisons between groups: penicillin vs. cefazolin (*p* = 0.077), penicillin vs. clindamycin (*p* = 0.246), cefazolin vs. clindamycin (*p* = 0.092).

**Table 2:** Maternal negative GBS colonization after IAP.

Type of IAP	One Dose	Two Doses or Above	Total	*p*
Penicillin	5/8 (62.5%) ^b^	62/75 (82.7%) ^b^	67/83 (80.7%) ^b^	0.366
Cefazolin	115/123 (93.5%)	82/84 (97.6%)	197/207 (95.2%)	0.304
Clindamycin	1/1 (100.0%)	3/4 (75.0%)	4/5 (80.0%)	0.800
Total	121/132 (91.7%) ^a^	147/163 (90.2%) ^a^	268/295 (90.8%) ^a^	0.661

Data were presented as negative counts/total counts (%); p: comparisons between one dose and two doses or above; ^a^, *p* < 0.05 in comparisons between penicillin, cefazolin and clindamycin; ^b^, *p* < 0.017 in comparison with cefazolin.

**Table 3:** Antimicrobial susceptibility based on the broth microdilution method.

Antimicrobial	MIC Range (µg/mL)	MIC50 (µg/mL)	MIC90 (µg/mL)	Sensitive (%)	Intermediate Sensitive (%)	Resistant (%)
Penicillin	≤0.12	≤0.12	≤0.12	100.0%	0	0
Ampicillin	≤0.25	≤0.25	≤0.25	100.0%	0	0
Clindamycin	≤0.25–≥8	≤0.25	≥8	78.6%	0	21.4%
Erythromycin	≤0.25–≥8	≤0.25	≥8	50.0%	10.0%	40.0%
Vancomycin	≤0.5	≤0.5	≤0.5	100.0%	0	0
Levofloxacin	0.5–≥8	1	≥8	84.4%	0	15.6%
Quinupristin-Dalfopristin	≤0.25–0.5	≤0.25	0.5	100.0%	0	0
Tetracycline	≤1–≥16	≥16	≥16	10.7%	0	89.3%
Linezolid	1–2	2	2	100.0%	0	0

CLSI does not provide breakpoints of cefazolin, cefepime, ceftibuten and ceftizoxime.

**Table 4:** Maternal and neonatal outcomes among the IAP groups.

Outcomes	Total	Penicillin (n = 83)	Cefazolin (n = 207)	Clindamycin (n = 5)	*p*
Maternal					
Vaginal delivery	279 (94.6%)	76 (91.6%)	199 (96.1%)	4 (80.0%)	-
Emergency cesarean section	16 (5.4%)	7 (8.4%)	8 (3.9%)	1 (20.0%)	0.071
Prelabor rupture of membranes	71 (24.1%)	17 (20.5%)	54 (26.1%)	0	0.345
Intrauterine infection	2 (0.7%)	2 (2.4%)	0	0	0.112
Chorioamnionitis	9 (3.1%)	4 (4.8%)	5 (2.4%)	0	0.386
Neonatal					
Low birth weight	4 (1.4%)	0	4 (1.9%)	0	0.609
NICU admission	16 (5.4%)	6 (7.2%)	10 (4.8%)	0	0.551
5 min Apgar score < 7	1 (0.3%)	0	1 (0.5%)	0	0.714
Neonatal early-onset sepsis	1 (0.3%)	1 (1.2%)	0	0	0.298

Data were presented in counts and percentages.

## Data Availability

The data presented in this study are available on request from the corresponding author.
